# Specific effects of working memory training on the reading skills of Chinese children with developmental dyslexia

**DOI:** 10.1371/journal.pone.0186114

**Published:** 2017-11-16

**Authors:** Juanhua Yang, Jun Peng, Dake Zhang, Liling Zheng, Lei Mo

**Affiliations:** 1 Center for the Study of Applied Psychology and School of Psychology, South China Normal University, Guangzhou, China; 2 Department of Educational Psychology, Rutgers University, New Brunswick, NJ, United States of America; 3 Guangdong Provincial Key Laboratory of Mental Health and Cognitive Science, South China Normal University, Guangzhou, China; Hangzhou Normal University, CHINA

## Abstract

Most research on working memory (WM) training for children with developmental dyslexia (DD) has focused on western alphabetical languages. Moreover, most of these studies used a combination of training tasks targeting a variety of WM components, making it difficult to determine whether WM training generates a general improvement in overall reading, or improves specific cognitive skills corresponding to the WM components that are targeted in training. We tested the general and specific effects of WM training on the reading skills of 45 Chinese children with DD, grades 3 to 5. In Experiment 1, the experimental group received a program targeting the verbal WM component; in Experiment 2, the experimental group was trained with a program targeting visuospatial WM. In both experiments the control group played a placebo video game. In Experiment 1, the experimental group outperformed the control group on the visual rhyming task, which is highly correlated with verbal WM. In Experiment 2, the experimental group outperformed the control group on the orthographic awareness test, which is highly correlated with visuospatial WM. Furthermore, in both Experiment 1 and Experiment 2, the experimental groups outperformed the control groups on the fast word naming test, which is highly related to both visuospatial WM and verbal WM. Results indicated that WM training improved specific reading-related cognitive skills that are highly correlated with the specific WM components that were the target of training.

## Introduction

At present, interventions for dyslexia are an important focus of WM training research. Dyslexia has been defined as a difficulty interpreting written language caused by core deficits in phonological awareness, independent of intelligence, educational level, and socioeconomic status [1–3]. Dyslexia is the most common developmental disorder, accounting for about 5%-15% of the school-age population in American [4]. Dyslexia is just as common among Chinese children [5], suggesting that the disorder is evident in children learning to read non-alphabetic as well as alphabetic languages.

The World Mental Health Organization divides dyslexia into acquired dyslexia and developmental dyslexia [6]. The former refers to dyslexia due to disease or brain injury, whereas the latter refers to a developmental problem that occurs during an individual’s development and is persistent throughout life. Those with developmental dyslexia (hereinafter referred to as DD, or dyslexia) show no difference in their general intelligence, motivation, living environment, or educational conditions, compared to their typically developing peers [7]. They have no obvious vision, hearing, or neural system disorders; however, their reading performance is significantly lower than what is expected for their age and grade [8].

### Working memory and language processing

WM has been described as a dynamic processing system with limited capacity that temporarily stores and processes information [9, 10]. It is the basis of many other cognitive processes, such as language comprehension, problem solving, and fluid intelligence [11, 12]. WM is also considered to be a basic ability that individuals must use to acquire new knowledge and skills [13–15]. It is highly correlated with vocabulary acquisition [16], language comprehension [17] and reading abilities [15]. Baddeley & Hitch [18] proposed a WM model with one core component (i.e., the central executive) and two subordinate components (i.e., the phonological loop and visuospatial sketchpad). Later, a third subordinate component—the episodic buffer—was added [19]. The *central executive* is responsible for coordinating information from the subordinate components by retrieving and manipulating information, and it directs resources to storage components [18]. The *phonological loop* is responsible for processing speech information. It plays an important role in language acquisition [20], including learning vocabulary[16] and learning a second language [21]. *The visuospatial sketchpad* is responsible for processing visual and spatial information, and is thought to be important for abilities such as nonverbal intelligence and orthographic awareness [22]. *The episodic buffer* is capable of binding information from the other subordinate components, and from long-term memory, into a unitary episodic representation [19]. The episodic buffer is associated with children's vocabulary recognition skills, and the ability to bind information in the episodic buffer grows with age [23].

In general, the results of previous studies have shown that the WM impairment experienced by those who have dyslexia is manifested not only in the language component of WM (the phonological loop), but also in the visuospatial component (the visuospatial sketchpad) and the central executive. Adolescents with dyslexia have been shown to exhibit a higher error rate and slower reaction times in the Wisconsin card sorting test, which is related to central executive functioning [24]. Children with special verbal deficiencies, although they have normal nonverbal intelligence, hearing, and pronunciation, show a reduced ability to perform non-word repetition tasks that assess functioning of the phonological loop [25], and children with dyslexia underperform in the rhyme judgment task, which is related to the phonological loop [26]. Studies have also shown that children with dyslexia perform worse than those with typical reading ability in tasks that require use of the visuospatial sketchpad [27]. As the episodic buffer is a relatively new concept, there have been few studies on it. One study showed that there was no significant difference between the typically developing reading group and the poor reading group in the episodic buffer task [28].

### Working memory and dyslexia

A large number of studies have shown that dyslexia is associated with poorer WM [29–31]. Compared to children with typical reading ability, children with dyslexia are poor at complex WM span tasks [32, 33] and tasks related to the central executive of WM [34]. Many studies have reported that children with dyslexia appear to have reduced ability during verbal short-term memory span tasks [35–37]. WM deficits in those with dyslexia appear to be due not only to deficits in verbal WM (i.e., the phonological loop), but also to deficits in the visuospatial WM (i.e., the visuospatial sketchpad) and the central executive [35, 37, 38].

Similar results were obtained in studies of Chinese children with dyslexia, who have been shown to have difficulty storing information in the phonological loop and the central executive [39]. Other research using a visual rhyming task suggested that Chinese children with dyslexia have specific deficiencies in the repetition function of the phonological loop [30]. These results are consistent with other evidence showing that the correlation between WM and language comprehension score was.72-.90 [40], suggesting that WM ability is an effective indicator of learning difficulties in the Chinese population.

Brain imaging studies have also shown a link between dyslexia and deficiencies in WM ability. Wu, Yao & Yu [41]used functional near-infrared optical imaging (fNIRI) to compare brain activation between children with and without dyslexia during the paced serial addition test (PSAT). They found that children with dyslexia had less activation in the left prefrontal cortex (a key region related to WM ability). The same results were obtained with an fMRI study [42]. Other studies [43, 44] also suggested that children with dyslexia have lower WM ability than those without dyslexia.

### The effect of WM training for dyslexia

Because of the important role of WM in basic cognitive process and the advanced functional learning of individuals, WM training has been paid more and more attention in various fields. Jaeggi, Buschkuehl, Jonides & Perrig [45]found that WM could be improved through training and that this training effect is transferable, meaning that it can also improve other cognitive abilities.

Previous research has found that children with dyslexia can benefit from early orthographic training and phonetic spelling training [46, 47]. Because many studies have suggested that those with dyslexia are indeed deficient in WM [24, 26, 27, 35, 37, 38], researchers have also begun to focus on improving dyslexia through WM training. Temple et al., [42]used fMRI to investigate brain activation in those with dyslexia during a rapid acoustic processing task, a measure of rapid acoustic signal response. Compared to those without dyslexia, those with dyslexia showed less activation in the left prefrontal cortex. After completing a 33-day, 100 minutes per day, rapid continuous processing training associated with WM, the left frontal lobe activation of some participants with dyslexia increased, and their test scores on the rapid acoustic processing task improved, indicating that people with dyslexia who have rapid hearing signal response deficits can be helped through WM training.

Horowitz-Kraus & Breznitz [48]had adults with and without dyslexia use the CogniFit Personal Coach (CPC) training program. CPC is a software program designed for integrated WM training that includes three WM span training tasks: language, visual, and linguistic-visual. The results showed that both those with and without dyslexia made significant progress in the WM span task after training. Similarly, Shiran & Breznitz [49]found that using WM training improved reading ability and increased the amplitude of the ERP P300 component (an indicator of reading ability, with higher amplitude indicating better reading ability) in those with dyslexia compared to those without dyslexia.

Tilanus, Segers & Verhoeven [50]used a 12-week phonological training to intervene in Dutch children with dyslexia. They found that compared with children without dyslexia, the children with dyslexia made more progress in tasks associated with reading (grapheme-phoneme correspondences, decoding words and pseudowords, rapid automatized naming).

Though there have been many studies on using WM training to improve dyslexia-related deficits in alphabetic languages, only one study has done so with the non-alphabetic language of Chinese. In that study, Luo et al., [30]used visuospatial WM training, phonological WM training, and a central executive inhibition function training program (Flanker task) to train Chinese children with dyslexia for five weeks. They used WM tasks and a reading skills test before and after training to explore the effect of the training. The results showed that the training intervention not only promoted performance in the WM task, but also significantly increased scores on the reading skills test (including a visual rhyming task and a one minute fast reading test).

Most of the above studies used the n-back paradigm for WM training, which was first employed by Jaeggi et al [45]. This method requires the participants to process a series of stimuli and to determine whether the current stimulus is the same as one presented a specific number of trials (*n*) back in the sequence. The level of difficulty (n-back) is interactively adjusted depending on the performance of the participants. When n becomes larger, the difficulty increases, resulting in changes in accuracy rate and reaction time [51].

In general, research has shown that WM training for dyslexia can not only improve performance of skills that were the target of training, but can also improve reading skills, showing an effect of training migration in reading speed tasks and reading comprehension tasks. However, research on the effectiveness of WM training in people with dyslexia is mainly focused on dyslexia in the West. The current study builds on the very limited literature on WM training for dyslexia in China [30].

Meanwhile, it should be pointed out that the current study on the improvement of dyslexia using WM training aims to solve an important problem concerning the specificity of WM training. Previous studies have shown that WM impairment in people with dyslexia is manifested not only in the phonological loop, but also in the visuospatial sketchpad and the central executive. However, most of the current improvement seen in dyslexia after WM training occurs when a variety of tasks are used to train participants (i.e., mixed training tasks) [30, 48]. Therefore, it is difficult to determine whether the effect of WM training on dyslexia is specific or general, which makes it important to further explore this question.

### The current study

Based on the previous research, the current study conducted two experiments. Experiment 1 and Experiment 2 tested the improvement of reading skills in Chinese children with dyslexia using phonological WM training and visuospatial WM training, respectively. Together, these experiments tested the effectiveness of WM training’s improvement of deficits seen in Chinese children with dyslexia, and also tested whether each type of WM training (phonological or visuospatial) had a specific effect on the reading-related cognitive skills associated with the target of training.

If phonological WM training (Experiment 1) or visuospatial WM training (Experiment 2) improves the reading ability of Chinese children with dyslexia, then one could conclude that the WM training is indeed effective in improving reading skills in this population. In terms of the specificity of the training effect, we would expect to see increases in the specific skills that were the target of the specific type of WM training. Specifically, we would expect to see children’s phonological loop awareness to be enhanced after phonological WM training (Experiment 1), and children’s orthographical awareness to be improved after visuospatial WM training (Experiment 2).

## Experiment 1

### Method

### Participants

We used four standards to select the children with developmental dyslexia from a primary school in Guangzhou, China: (1) they had normal nonverbal intelligence measured by Raven's standard Progressive Matrices (SPM); (2) Pupil Rating Scale Revised-Screening for Learning Disability (PRS) scores were less than 60; (3) Chinese language scores were lower than 95% of students in the same grade; and (4) Volume of Lexical Acquisition (VLA) was 1.5 standard deviations below average for their grade level [52]. Standards (2), (3), (4) were based on information provided by their teachers. The research was approved by the Human Research Ethics Committee at South China Normal University, and the teachers and parents of participating children provided informed written consent.

Twenty-five children with developmental dyslexia were selected and randomly divided into an experimental group (9 males and 4 females) and a control group (8 males and 4 females). One participant in the experimental group was excluded for repeated absences from the training, and one participant was excluded from the control group for illness. The data from the remaining 23 children were used in the experiment, with 12 participants in the experimental group (8 males and 4 females; 9.71±.78 years old) and 11 participants in the control group (8 males and 3 females; 9.72±.45 years old). There were no significant differences in the intelligence, age, gender, or VLA between the two groups (*p*s > .05).

### Design and materials

This experiment was a 2 (Test: pretest, posttest) x 2 (Group: experimental, control) mixed design. The experimental group completed single phonology n-back WM training, and the control group played the placebo Idiom King video game, which focuses on verbal skills. The experiment was double-blind, meaning that the teachers who presented the training program or placebo video game did not know the experiment’s purpose. Similarly, pretest and posttest were conducted by the same tester without knowing the group division. Experiment 1 focused on the phonological loop component of WM.

#### Experimental group WM training program

**Fig 1:**
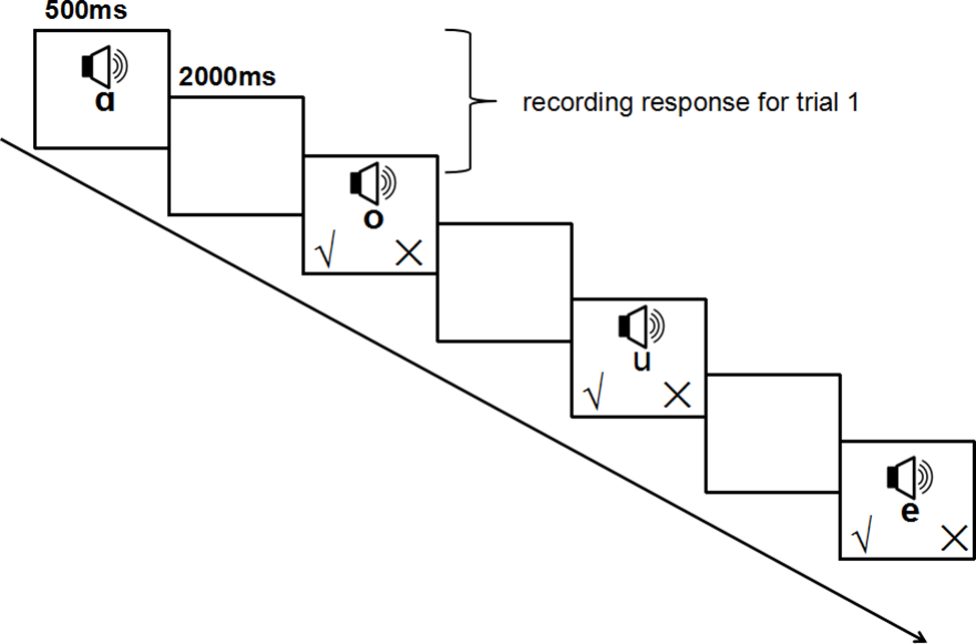
Illustration of 1-back phonological WM training of Experiment 1.

We used a self-compiled WM training program run on tablet computers to train the experimental group. This program is similar to the classic paradigm but easier to be understood by students from grades 3 to 5. The training used 9.7-inch screen tablet computers with a screen resolution of 1024 * 768. There were eight levels of difficulty in the single phonology n-back WM training program (i.e., 1-back to 8-back, see Fig 1 for 1-back training program). Each level had 15 + n trials, and each auditory stimulus was presented for 500ms. Six Chinese vowels ("ɑ," "o," "e," "i," "u," and "ü") were presented randomly across trials. Participants had 2500ms to determine at the end of each trial whether the current phonology was the same as the phonology n trials ago by pressing the buttons on the screen (“**√**” for same, “**×**” for different). For example, in a 1-back task, the participant heard “ɑ” first, and then heard “o,” and so the determination should be “different” for the two vowels. Of the trials, 10 + n would be accurately judged as different, and 5 would be accurately judged as same.

#### Control group video game training program

The control group played the Idiom King video game on the same tablet computers. The game required the player to quickly and accurately choose the word that completed a four-word idiom from three words displayed on the screen. The difficulty level was interactively adjusted. When the correct answer was selected, the difficulty increased, and the time provided for choosing the next word was shortened. If the incorrect answer was selected, the difficulty decreased, and the time provided was lengthened. Once the number of errors reached 4, the round of the game was terminated.

#### Reading skills test

The reading skills test consisted of three parts: the orthographic awareness test, the phonological awareness test, and the fast word naming test. The three tests were conducted once before and once after the training, and the accuracy rate and reaction time were recorded. The tests were carried out with computers located in the computer room of the primary school. The computers had 17-inch screens with a screen resolution of 1024 * 768.

**Fig 2:**
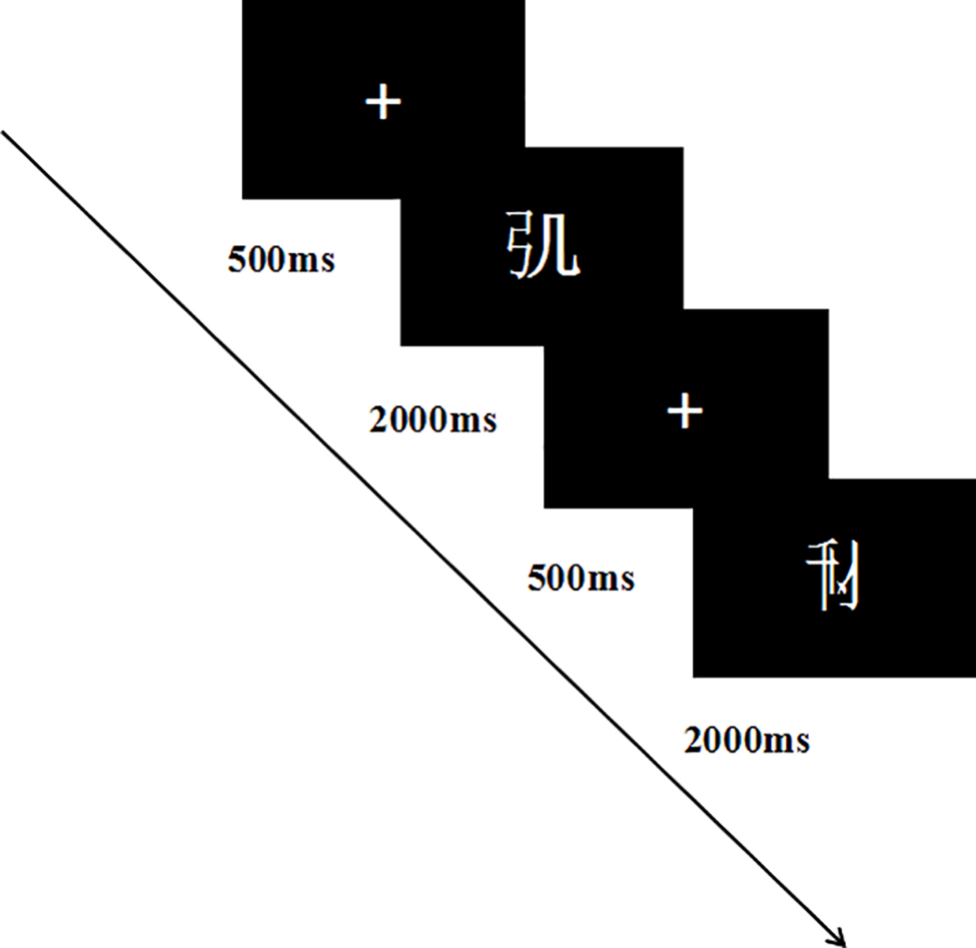
Illustration of orthographic awareness test.

Orthographic Awareness Test: The materials for the orthographic awareness test were adapted fromHo, Chan, Tsang & Lee [53]. Participants were tested on computers using E-prime programming (see Fig 2). The test consisted of 35 pseudowords (complied with the rules of Chinese orthography, but were not real characters, e.g. 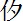) and 35 nonwords (violated Chinese orthography rules, and were not real characters, e.g. 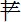), and the number of strokes was matched between the pseudowords and the nonwords. There were six practice trials before the start of the formal test. Each trial contained only one “word” and they were presented at random. Participants were instructed as follows: “If you think it may be a Chinese word, please press 'F', otherwise press 'J'.” Each word was presented for 2000ms, and children needed to respond within the 2000ms. The inter-stimulus interval was 500ms. After 35 trials the children were given a break to rest for as long as they needed, with the instruction to press "P" when they were ready to continue. We used parallel lists of items for the pretest and posttest, and the number of strokes was matched for the two versions.

**Fig 3:**
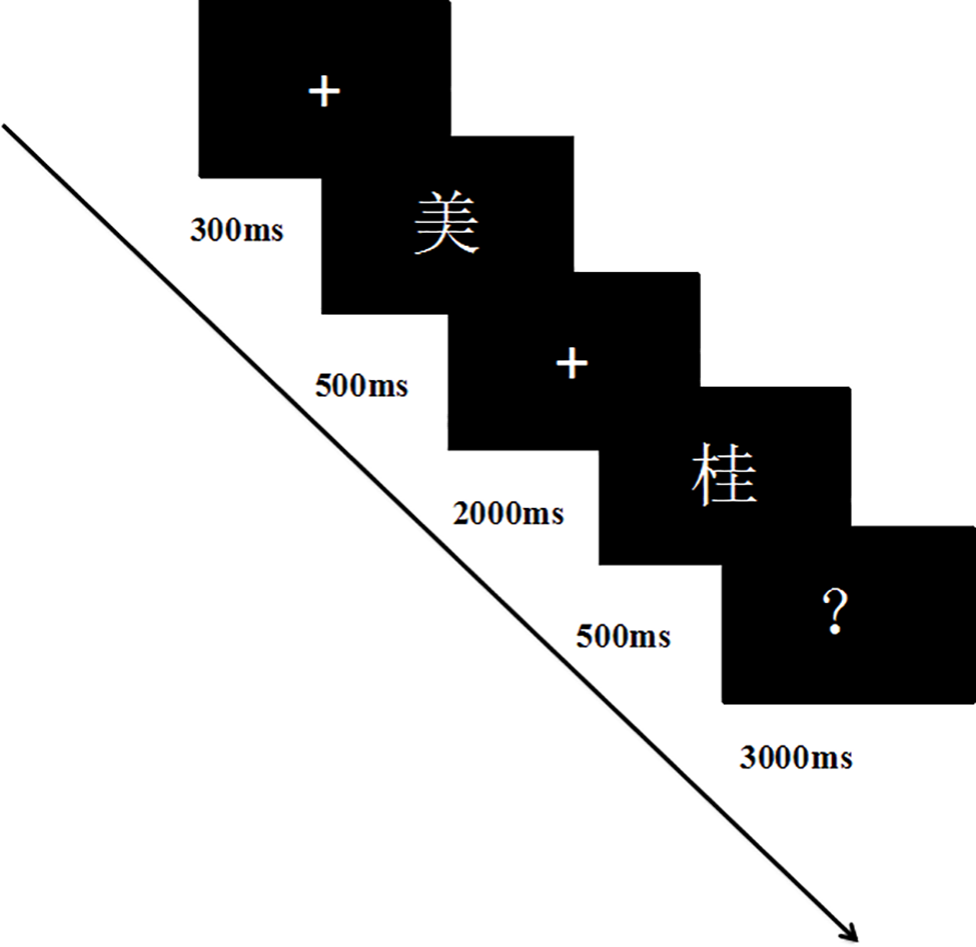
Illustration of visual rhyming task.

Phonological Awareness Test: A visual rhyming task was used to test phonological awareness based on the paradigm used byGrossi, Coch, Coffey-Corina, Holcomb & Neville [54]. Participants were tested on computers using E-prime programming (see Fig 3). They were instructed to determine if the vowel between the priming word and the target word was the same. They had 3000ms to respond by pressing “F” if it was the same or “J” if it was different. Both the priming word and the target word were presented for 500ms. The interval between them was 2000ms. There was a total of 40 trials (20 trials with the correct answer "same", 20 trials with the correct answer "different") presented in a random order. We used parallel lists of items at pretest and posttest, and the number of strokes was matched for priming words and target words.

Fast Word Naming Test: Fast word naming is related to phonological awareness as well as orthographical awareness. We revised the materials for the fast word naming test from a previous study byLiao, Georgiou & Parrila [55], which resulted in a final total of 40 words with two characters for each (e.g., 花朵, meaning flower). The participants were asked to read these words as quickly and accurately as possible, and the experimenter recorded the time with a stopwatch. Parallel lists of items were used for the pretest and posttest, and the number of strokes and the word frequencies were matched for the words and characters included in each list.

#### Attitude and motivation questionnaire

To make sure that there was no difference in the attitude and motivation between the two groups of participants, we conducted the attitude and motivation questionnaire. Items for the attitude and motivation questionnaire were compiled by the researchers based on the literature. There were 5 questions, including interest, level of effort, and self-achievement evaluation. Participants responded using a 5-point Likert scale. In order to facilitate children’s understanding, the questions and answer choices were illustrated by the teachers. The internal consistency coefficient of the questionnaire in this study was.764. Both the experimental and control groups were tested after the training was completed.

### Procedures and scoring

#### Procedure and scoring for experimental group

Before completing training, participants completed the reading skills pretest. Each participant then received 10 sets (one set consists of 15 + n trials) of training every day for 15 days (5 days a week for 3 weeks), with each day’s training taking about 15 minutes. In order to ensure that the children were energetic, the daily training was carried out from 14:15 to 14:30 after they had a break. The students were trained in the school computer classroom that was familiar to them. The classroom was well lighted, well ventilated, and relatively quiet. Each tablet computer used in the training was marked with the student’s name and number. Before the start of daily training, the experimenter arranged the desks and chairs and debugged the tablet computers. The brightness, resolution, and headset volume were the same for all the tablet computers. The participants were offered stationery as a reward from the experimenter after daily training.

Each participant in the experimental group was trained starting from the 1-back task (i.e., the first level). For each level, if the participant had 3 or fewer wrong trials, they were able to move on to the next level; if they had 4 or more wrong trials, they stayed at that level and retrained. They were given two chances to retrain. If they were still unable to pass the level, they moved to a lower level (If they were having trouble passing the first level, they remained at that level.). Each participant was trained for 10 sets a day, and the last level they ended on for each day was recorded. During the next training session, they started from the level where they left off in the previous training session. The participants’ daily performance in WM training was determined by the level that they ended on. For example, if on the first day, a participant ended on the second level, then the result of their first day was recorded as 2. If the next day the participant ended on the fourth level, the result recorded for that day was 4. After completing all training, participants completed the reading skills posttest and the attitude and motivation questionnaire.

#### Procedure and scoring for control group

Before they began playing the placebo video game, control group children completed the reading skills pretest. The control group played the game in the same place and for the same amount of time as the experimental group. Performance was scored in the same way as it was for the experimental group, based on what level of the game they had attained at the end of each session. The participants were also offered stationery as a reward from the experimenter after daily training. After all sessions of playing Idiom King, participants completed the reading skills posttest and the attitude and motivation questionnaire.

## Analyses and results

All data were input and processed in SPSS (Statistical Product and Service Solutions) 13.0 for Windows. SPSS is a professional statistical program for social sciences, and it is widely used in data analysis.

### Phonological WM training results

**Fig 4:**
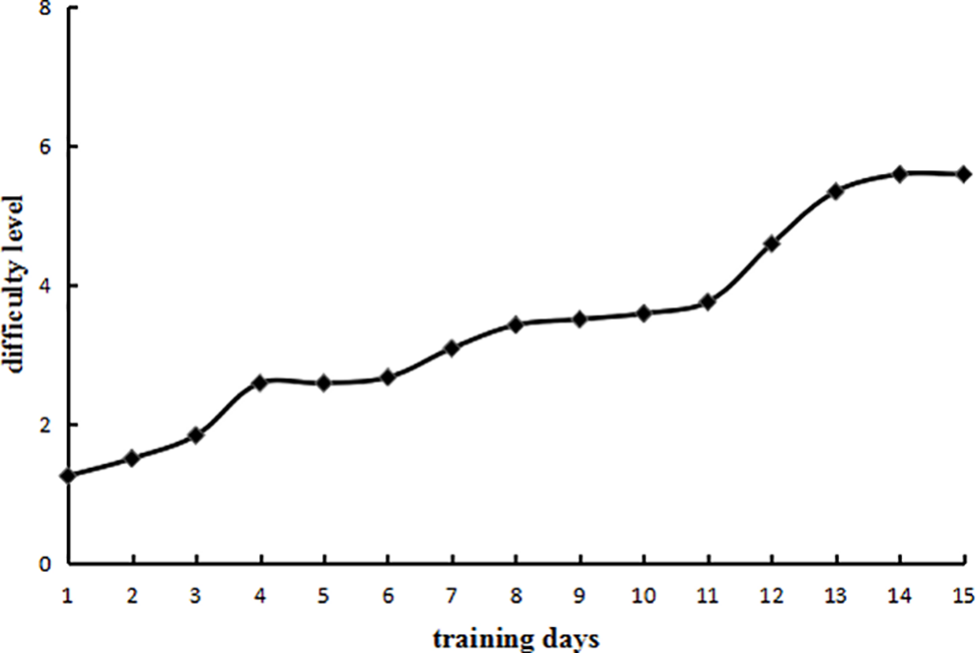
Experimental group WM training results of Experiment 1.

The average level (1-back to 8-back) reached by participants in the experimental group after daily training over the 15 days is shown in Fig 4. To demonstrate that the experimental group had completed the training and progressed as required, we conducted a paired samples t-test to compare the average of the levels reached on the first two days of training and the average of the levels reached on the last two days of training. The difference was significant (*t* (11) = 5.52, *p* < .001, Cohen’s *d* = 1.59). Thus, the participants were better able to complete the task at the end of training than at the beginning of training, suggesting that the WM training with the n-back task was effective.

### Reading skills test results

Table 1 shows the performance of the two groups on the phonological awareness test (visual rhyming task), the orthographic awareness test, and the fast word naming test.

**Table 1:** Results for Experiment 1 on the reading skills tests before and after WM training.

	pretest		posttest	
	Experimental group	Control group	Experimental group	Control group
	M(SD)	M(SD)	M(SD)	M(SD)
**Visual rhyming task**				
Accuracy rate	.39(.11)	.41(.11)	.60(.16)	.45(.19)
Reaction time(ms)	1169.65(489.26)	877.12(235.37)	1078.40(455.80)	1073.15(433.43)
**Orthographic awareness test**				
Accuracy rate	.46(.20)	.55(.19)	.53(.21)	.65(.21)
Reaction time(ms)	640.71(174.28)	677.06(155.84)	699.73(135.85)	782.08(144.86)
**Fast word naming test** (s)	36.16(11.09)	32.20(7.39)	30.71(6.61)	34.26(6.77)

Note. Letters “ms” in parentheses is short for millisecond, and “s” in parentheses is short for second.

#### Visual rhyming task results

A 2 (Test: pretest, posttest) x 2 (Group: experimental, control) mixed between-within subjects repeated measures ANOVA was conducted for the accuracy rate on the visual rhyming task. The results showed that the interaction between the two factors was significant (*F*(1,21) = 8.63, *p* < .01, η^2^_p_ = .30). The main effect of Test was also significant (*F*(1,21) = 16.73, *p* < .01, η^2^_p_ = .44). The main effect of Group was not significant (*F*(1,21) = 1.50, *p* = .23, η^2^_p_ = .07).

**Fig 5:**
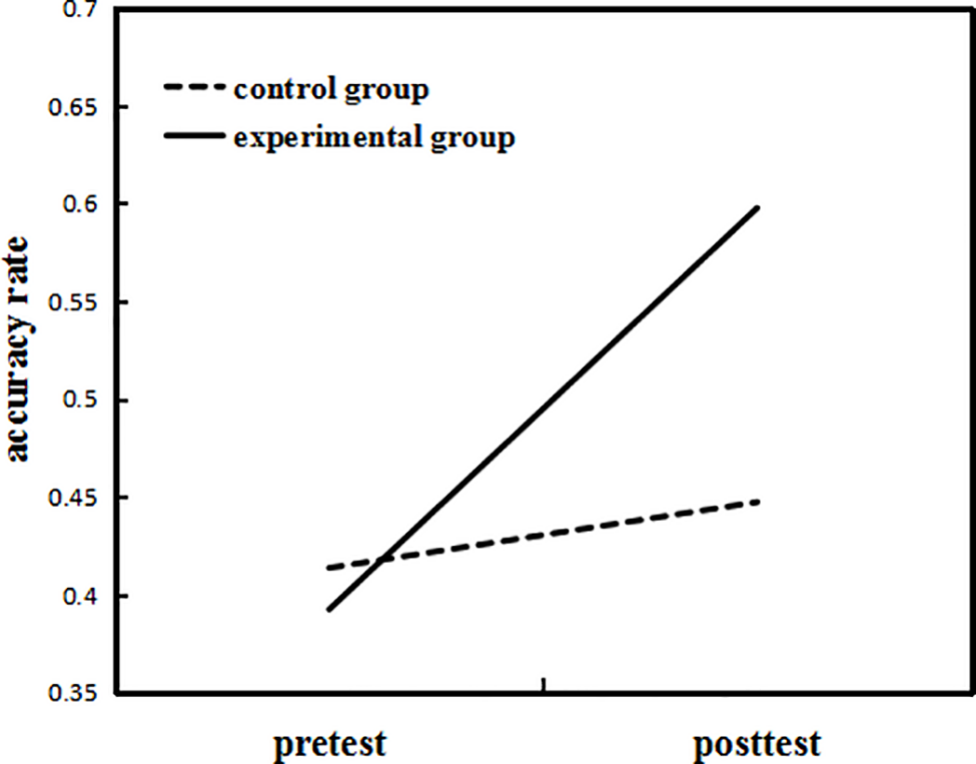
Accuracy rate on the visual rhyming task for the two groups of Experiment 1.

The Group x Test interaction effect was further analyzed using simple effects analysis. First we compared the experimental and control group scores for accuracy rate at pretest and posttest. The results showed that there was no significant difference between the two groups on the pretest (*F*(1,21) = .22, *p* = .65, *η*^*2*^_*p*_ = .01). There was, however, a significant difference between the two groups on the posttest (*F*(1,21) = 4.33, *p* = .05, *η*^*2*^_*p*_ = .17), with the accuracy rate in the experimental group being higher than that of the control group. We then compared the differences between pretest and posttest of the two groups separately. We found that for the experimental group, the accuracy rate on the posttest was much higher than that on the pretest (*F*(1,21) = 25.82, *p* < .001, *η*^*2*^_*p*_ = .55), but there was no significant difference for the control group (*F*(1,21) = .64, *p* = .43, *η*^*2*^_*p*_ = .03). The results are shown in Fig 5.

A 2 (Test: pretest, posttest) x 2 (Group: experimental, control) mixed between-within subjects repeated measures ANOVA was conducted for the reaction time on the visual rhyming task. The results showed that the interaction between the two factors was not significant (*F*(1,21) = 2.05, *p* = .17, *η*^*2*^_*p*_ = .09), nor were the main effects (*p*s > .05).

The above results showed that the experimental group performed better on the phonological awareness test (visual rhyming task) after the phonological WM training, which suggests that phonological WM training can improve the phonological awareness of Chinese children with dyslexia.

#### Orthographic awareness test results

A 2 (Test: pretest, posttest) x 2 (Group: experimental, control) mixed between-within subjects repeated measures ANOVA was conducted for the accuracy rate on the orthographic awareness test. The results showed that the interaction between the two factors was not significant (*F*(1,21) = .08, *p* = .79, *η*^*2*^_*p*_< .01). The main effect of Group was also not significant (*F*(1,21) = 2.10, *p =* .16, *η*^*2*^_*p*_ = .10), nor was the main effect of Test (*F*(1,21) = 3.23, *p* = .09, *η*^*2*^_*p*_ = .13).

A 2 (Test: pretest, posttest) x 2 (Group: experimental, control) mixed between-within subjects repeated measures ANOVA was conducted for the reaction time on the orthographic awareness test. The results showed that the interaction between Test and Group was not significant (*F*(1,21) = .36, *p* = .56, *η*^*2*^_*p*_ = .02), nor was the main effect of Group (*F*(1,21) = 1.36, *p* = .26, *η*^*2*^_*p*_ = .06). However, the main effect of Test was significant (*F*(1,21) = 4.46, *p*< .05, *η*^*2*^_*p*_ = .18), with the reaction time being longer in the posttest than in the pretest for both groups.

The above results showed that the phonological WM training did not improve the orthographical awareness of the participants, although it did improve the phonological awareness.

#### Fast word naming test results

A 2 (Test: pretest, posttest) x 2 (Group: experimental, control) mixed between-within subjects repeated measures ANOVA was conducted for the reaction time of the two groups. The results showed that the interaction between Group and Test was significant (*F*(1,21) = 7.01, *p* = .02, *η*^*2*^_*p*_ = .25). Neither of the main effects was significant (*p*s>.05). The interaction effect was further analyzed with simple effects analysis. A significant difference was found between the pretest and the posttest for the experimental group (*F*(1,21) = 7.71, *p* = .01, *η*^*2*^_*p*_ = .27), with the reading time in the posttest significantly less than that of the pretest. The difference was not significant for the control group (*F*(1,21) = 1.01, *p* = .33, *η*^*2*^_*p*_ = .05).

These results showed that to some extent, phonological WM training improved the speed of fast word naming for Chinese children with dyslexia. The ability of fast word naming is assumed to be related both to phonological awareness and orthographical awareness. Thus, the training effect was also reflected in the fast word naming task.

Overall, the results of Experiment 1 showed that phonological WM training significantly improved the phonological awareness of the experimental group, but not orthographic awareness. Participants in the experimental group also performed better on the fast word naming test, which is partly phonological in nature.

### Attitude and motivation questionnaire results

We calculated the total score on the attitude and motivation questionnaire, and an independent samples t-test was conducted to compare the overall scores of the two groups. The result showed that there was no significant difference between the two groups (*t*(21) = .68, *p* = .50, Cohen's *d* = .28). This suggests that the improvement in phonological awareness was due to the phonological WM training and not due to motivation or attitude levels.

## Experiment 2

Experiment 1 tested the effect of phonological WM training on Chinese children with dyslexia. It was found that phonological WM training improved phonological awareness and reading fluency but not orthographic awareness. Experiment 2 tested the effect of visuospatial WM training on Chinese children with dyslexia.

### Method

### Participants

The selection criteria were the same as in Experiment 1. The participants were selected from students in grades 3 to 5 at a primary school in Guangzhou, China. None had participated in Experiment 1. Twenty-four children with dyslexia participated in the experiment and were randomly divided into an experimental group (9 males and 3 females) and a control group (8 males and 4 females). One of the participants in the experimental group was excluded for repeated absences from the training, and one participant was excluded from the control group for not completing the posttest. The data from the remaining 22 children were used in the experiment, with 11 participants in the experimental group (8 males and 3 females; 9.97 ± .73 years old) and 11 participants in the control group (8 males and 3 females; 9.67 ± .61 years old). There were no significant differences in intelligence, age, gender, or VLA between the two groups (*p*s>.05).

### Design and materials

The experimental design in Experiment 2 was the same as in Experiment 1.

#### Experimental group WM training program

**Fig 6:**
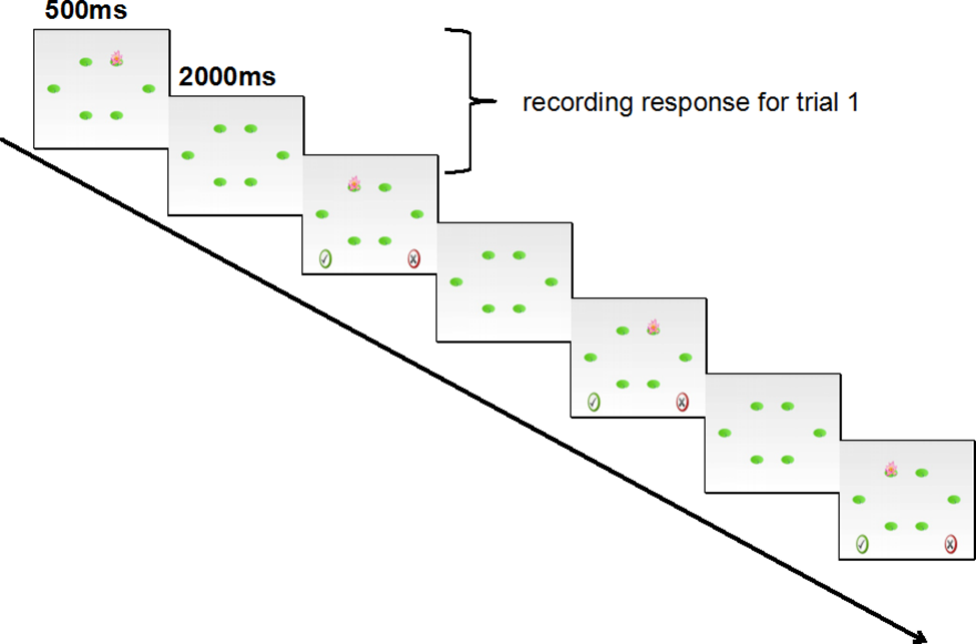
Illustration of 1-back visuospatial WM training of Experiment 2.

The training procedure was nearly the same as in Experiment 1, except for the stimuli. In Experiment 1, we used single phonology in the n-back paradigm. In Experiment 2, a single visuospatial n-back paradigm (see Fig 6) was used. In the training, six spatial positions of a circular arrangement were presented on the tablet screen. The target object would appear in a random spatial position for 500ms. The participants had 2500ms to indicate whether the current target was in the same position as the target n trials ago by pressing “**√**”if it was the same and “**×**” if it was different. There were a total of 15 + n trials for each level(10 + n in which the correct answer was “different” trials and 5 in which the correct answer was “same”) presented in a random order.

#### Control group video game training program

The control group was trained with the computer game Pull the Carrot on the same tablet computers. The game was downloaded from the web. It required the player to quickly and accurately slide their finger upwards to the position where an object appeared. Targets appeared continuously and quickly, and the participants needed to focus their attention within a limited period of time to achieve good results. The difficulty level was interactively adjusted. When the carrot was pulled, the difficulty increased, and the carrot provided for pulling was increased. If the carrot was not pulled, the difficulty decreased, and the carrot provided for pulling was decreased. Once the number of errors was greater than or equal to 4, the round of the game was terminated. The child’s score was the final level for that day.

Experimental apparatuses and data processing methods were the same as in Experiment 1.

## Analyses and results

All data were also input and processed in SPSS 13.0 for Windows as Experiment 1.

### Visuospatial WM training results

**Fig 7:**
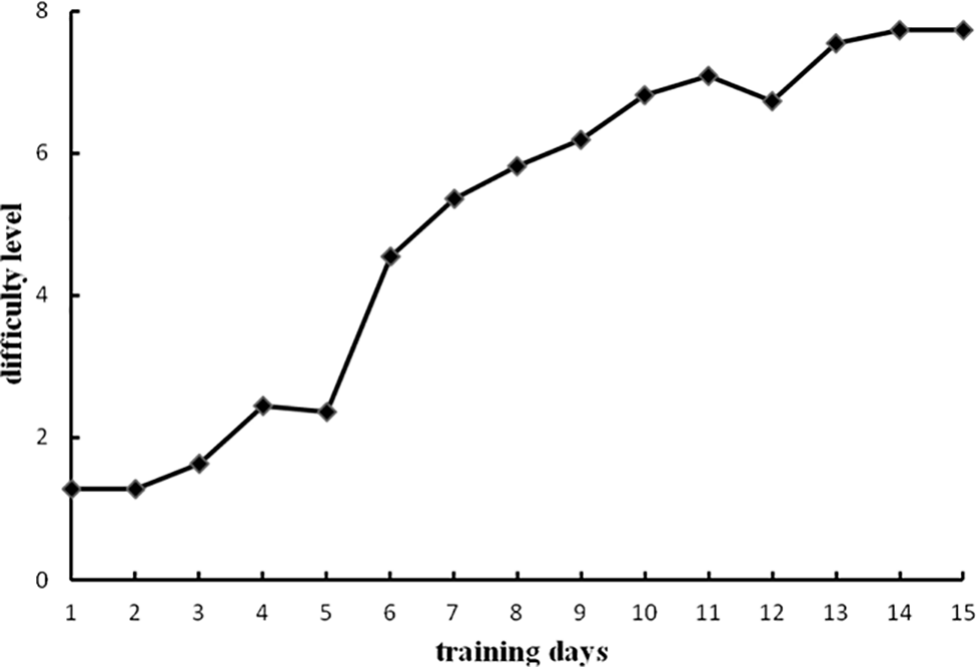
Experimental group WM training results of Experiment 2.

The average level reached by participants in the experimental group after daily training over the 15 days is shown in Fig 7. To demonstrate that the experimental group had completed the training and progressed as required, we conducted a paired samples t-test to compare the average of the levels reached on the first two days of training and the average of the levels reached on the last two days of training. The difference was significant (*t*(10) = 31.14, *p* < .001, Cohen’s *d* = 12.92). Thus, the participants were better able to complete the task at the end of training than at the beginning of training, suggesting that WM training with the n-back task was effective.

### Reading skills test results

Table 2 shows the performance of two groups on the visual rhyming task, the orthographic awareness test, and the fast word naming test.

**Table 2:** Results for Experiment 2 on the reading skills tests before and after WM training.

	pretest		posttest	
	Experimental group	Control group	Experimental group	Control group
	M(SD)	M(SD)	M(SD)	M(SD)
**Visual rhyming task**				
Accuracy rate	.40(.13)	.41(.10)	.41(.19)	.41(.10)
Reaction time(ms)	1095.55(403.23)	980.08(443.61)	1059.84(391.48)	1059.46(489.54)
**Orthographic awareness test**				
Accuracy rate	.62(.20)	.69(.22)	.82(.14)	.60(.16)
Reaction time(ms)	666.17(136.15)	750.93(134.23)	690.55(122.74)	666.32(152.36)
**Fast word naming test** (s)	36.32(7.90)	37.49(8.64)	32.63(7.96)	38.08(9.86)

Note. Letters “ms” in parentheses is short for millisecond, and “s” in parentheses is short for second.

#### Results of visual rhyming task

A 2 (Test: pretest, posttest) x 2 (Group: experimental, control) mixed between-within subjects repeated measures ANOVA was conducted for the accuracy rate on the visual rhyming task. The results showed that the interaction between the two factors was not significant (*F*(1,20) = .02, *p* < .88, *η*^*2*^_*p*_ < .01), nor were the main effects (*p*s>.05).

A 2 (Test: pretest, posttest) x 2 (Group: experimental, control) mixed between-within subjects repeated measures ANOVA was conducted for the reaction time on the visual rhyming task. The results showed that the interaction between the two factors was not significant (*F*(1,20) = .17, *p* = .69, *η*^*2*^_*p*_ < .01), nor were the main effects (*p*s > .05).

The above results suggested that visuospatial WM training did not improve the phonological awareness of the experimental group because the visuospatial WM training was related to the visuospatial sketchpad component of WM, not the phonological loop component. The phonological loop was not directly trained, thus, phonological awareness was not improved.

#### Orthographic awareness test results

A 2 (Test: pretest, posttest) x 2 (Group: experimental, control) mixed between-within subjects ANOVA was conducted for the accuracy rate on the orthographic awareness test. The results showed that the interaction between the two factors was significant (*F*(1,20) = 5.03, *p* = .04, *η*^*2*^_*p*_ = .20). The main effect of Group was not significant (*F*(1,20) = .72, *p* = .41, *η*^*2*^_*p*_ = .04), nor was the main effect of Test (*F*(1,20) = 3.14, *p* = .09, *η*^*2*^_*p*_ = .14).

**Fig 8:**
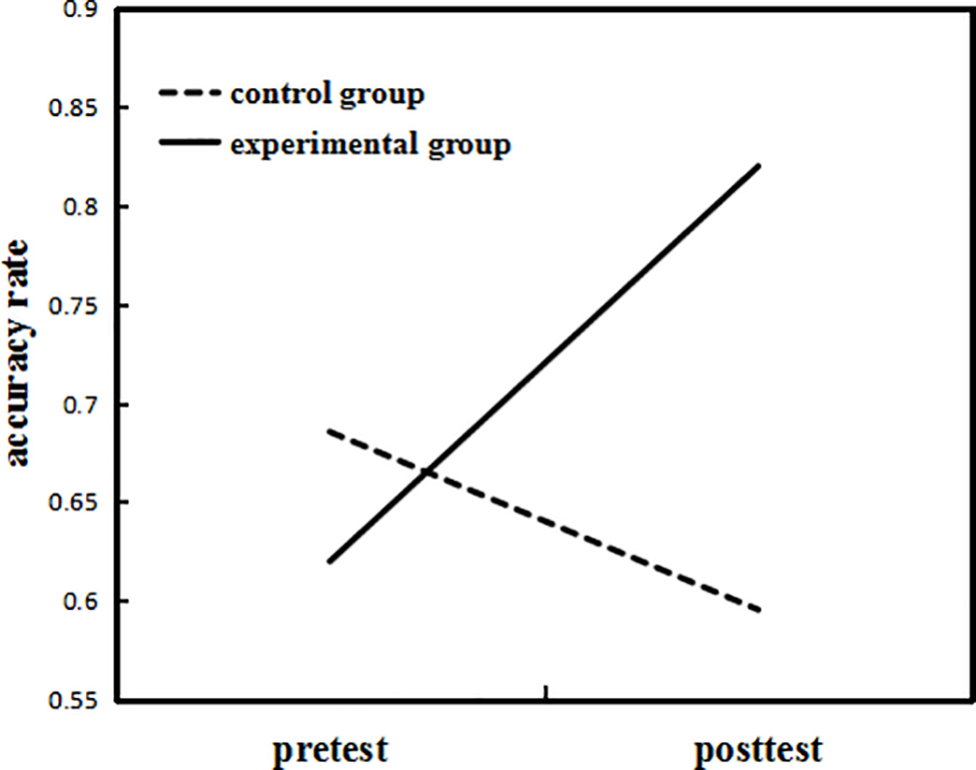
The accuracy rate on the orthographic awareness test for the two groups of Experiment 2.

The interaction effect was further analyzed using simple effects analysis. First we compared the accuracy rate in the experimental group and the control group at pretest and posttest. The results showed that there was no significant difference between the two groups on the pretest (*F*(1,20) = .51, *p* = .48, *η*^*2*^_*p*_ = .02). There was, however, a significant difference between the two groups on the posttest (*F*(1,20) = 12.47, *p* < .01, *η*^*2*^_*p*_ = .38), with the accuracy rate in the experimental group being higher than that of the control group. We then compared the differences between pretest and posttest of the two groups separately. We found that for the experimental group, the accuracy rate on the posttest was significantly higher than that on the pretest (*F*(1,20) = 4.78, *p* = .04, *η*^*2*^_*p*_ = .19), but there was no significant difference for the control group (*F*(1,20) = .97, *p* = .34, *η*^*2*^_*p*_ = .05). The results are shown in Fig 8.

A 2 (Test: pretest, posttest) x 2 (Group: experimental, control) mixed between-within subjects repeated measures ANOVA was conducted for the reaction time on the orthographic awareness test. The results showed that the interaction between the two factors was not significant (*F*(1,20) = 1.97, *p* = .18, *η*^*2*^_*p*_ = .09), nor were the main effect (*p*s>.05).

The above results showed that the experimental group performed better on the orthographic awareness test, but not on the phonological awareness test, after visuospatial WM training. This suggests that visuospatial WM training could improve the orthographic awareness of Chinese children with dyslexia.

#### Fast word naming test results

A 2 (Test: pretest, posttest) x 2 (Group: experimental, control) mixed between-within subjects repeated measures ANOVA was conducted for the reaction time of the two groups. The results showed that the interaction between Group and Test was marginally significant (*F*(1,20) = 3.82, *p* = .06, *η*^*2*^_*p*_ = .16). The main effects were not significant (*p*s > .05). The interaction effect was further analyzed using simple effects analysis. A significant difference was found between the pretest and the posttest scores for the experimental group (*F*(1,20) = 5.68, *p* = .03, *η*^*2*^_*p*_ = .22), with the reading time on the posttest significantly less than that on the pretest. The difference was not significant for the control group (*F*(1,20) = .15, *p* = .71, *η*^*2*^_*p*_ < .01).

The results of Experiment 2 showed that visuospatial WM training significantly improved the participants’ orthographic awareness (the visuospatial component of WM). Taken together, the results of Experiment 1 and Experiment 2 suggest that there is a specificity in WM training where only the function of the trained WM component is enhanced.

### Attitude and motivation questionnaire results

We calculated the total score of the attitude and motivation questionnaire, and an independent samples t-test was conducted to compare the overall scores of the two groups. The result showed that there was no significant difference between the two groups (*t*(20) = 1.26, *p* = .22, Cohen's *d* = .54). This suggests that the group difference in improvement in orthographic awareness was due to the visuospatial WM training and not due to group differences in motivation or attitude levels.

## Discussion

In recent years, many studies have provided evidence that short-term WM training could improve the reading ability of children with dyslexia. However, almost all of these studies have focused on alphabetic language, and it was not clear if training produced general or specific effects in reading skills. In this study, we used the phonological n-back paradigm and the visuospatial n-back paradigm to train the WM of Chinese students with dyslexia. In Experiments 1 and 2, we found that after 15 days of phonological WM training (Experiment 1) and visuospatial WM training (Experiment 2), the experimental group performed better on the phonological awareness test (Experiment 1) and the orthographic awareness test (Experiment 2) than before training. Furthermore, the experimental groups in Experiment 1 and 2 both performed better in the fast word naming test after training than before training. The above results indicate that WM training is also effective in improving the reading skills of Chinese dyslexia children. More importantly, this is the first study to explore whether WM training produces general or specific effects in reading skills of children with dyslexia. The results suggest that the type of WM training (phonological or visuospatial) is associated with the type of improvement in reading skills.

The above results indicate that the n-back paradigm of WM training was effective in improving the reading skills of Chinese children with dyslexia. More importantly, the positive effects of WM training were specific to the reading skills associated with the trained component. Specifically, the phonological n-back paradigm for WM training improved phonological awareness, whereas the visuospatial n-back paradigm for WM training improved orthographic awareness. The results of this study have expanded previous research on the use of WM training to improve the reading skills of those with dyslexia.

Most of previous studies on improving the reading skills of participants with dyslexia through WM training have consistently found that the training is effective [30, 42, 46–50]. However, these studies were focused on alphabetic-language readers with dyslexia. In the current study, native Chinese children with dyslexia were selected as participants, and the effectiveness of WM training in Chinese children with dyslexia was confirmed.

Evidence from behavioral and neuroscience studies has shown that people with dyslexia have deficits in their WM refresh function (i.e., the ability of dynamic memory updating) [37, 38, 56, 57]. In this study, we used the phonological n-back paradigm and the visuospatial n-back paradigm as WM training paradigms, which are considered by the academic community to promote the WM refresh function. These training paradigms produced significant training effects. However, neither earlier studies nor the current study employed other paradigms to investigate the training effects on reading skills. Therefore, in future research, it will be important to investigate additional available paradigms for effective training of WM or reading skills.

It is worth emphasizing that the WM training approaches used in this study did not improve reading skills by training the attention of the participants. Previous studies indicated that the participants’ attention increased after WM training [58–60], as well as 10 hours of video game training [61]. Control group participants in Experiment 1 and Experiment 2 played the Idiom King and Pull the Carrots games, which were also related to attention enhancement, but their reading skills were not significantly improved. This suggests that WM training improves the reading skills of children with dyslexia not through increasing their attention, but through improving their WM refresh function.

With regard to the specific effect of WM training, some previous studies tested whether different training tasks can be used to train different subcomponents of WM. The results have shown that phonological WM training can train the phonological loop system of WM, resulting in better performance on tasks that use the phonological loop. In one such study, the experimental group received music training and the control group completed natural science training. After 7 days of training, compared with the control group, the experimental group performed better on tasks related to the use of the phonological loop and the central executive [62]. Similarly, research by Schwarb, Nail & Schumacher [63]showed that visual WM training can improve participants’ short-term visual memory capacity.

There have been no studies thus far, however, that have directly tested the specific effect of WM training on the reading skills of participants with dyslexia. According to previous studies on WM deficits in those who have dyslexia, the deficits can be classified into three types: phonological WM deficit, visuospatial WM deficit, and mixed deficit [64]. Previous studies on WM training in those with dyslexia used a set of training tasks or, in the case of the CPC training program, a mixed task. Although the studies demonstrated the effectiveness of WM training in terms of improved reading skills of children with dyslexia, it is not clear whether this improvement was specific to certain aspects of reading. In contrast to the previous WM training research, the current study used one WM training task at a time (phonological n-back WM training in Experiment 1 and visuospatial n-back WM training in Experiment 2) to test the specific effects of different WM training. Thus, not only could we verify the effectiveness of WM training, but we could also test whether the WM training is specific.

The results of this study have shown that WM training can improve the reading skills of Chinese children with dyslexia, and they have important implications for the treatment of three subtypes of deficits associated with dyslexia (visuospatial processing deficit, phonological processing deficit, and mixed deficit). For example, when trying to improve visuospatial processing deficit, we could specifically train visuospatial WM, which is more direct and effective than using a mixed training task. The single WM training task needs less time than the mixed WM training task, which is especially important for some participants, such as those with attention deficit hyperactivity disorder (ADHD) who have difficulty in maintaining their attention for a long time.

It should be noted that this study has limitations: (1) Children in the experimental group and control group all had dyslexia; it may be useful in future research to include another control group of children without dyslexia, so that the implications of the results would be clearer. (2)The sample size is small,because it is very difficult to screen children with developmental dyslexia from the ordinary primary school. (3) We only conducted behavioral experiments to explore the effects of WM training, and more subtle methods like functional magnetic resonance imaging (fMRI) and event-related potentials (ERPs) may be useful in future studies. (4) Longitudinal research in the future should test whether the training effect is persistent, whether earlier intervention is more effective, and whether there is a critical period when intervention is most effective.

The current study provides more evidence to document the effects of WM training on reading in the context of a non-alphabetic language, and the first to decipher the general and specific effects of WM training focused on either the phonological or visuospatial components of reading. The findings provide a theoretical basis for the treatment of different subtypes of dyslexia. Compared to interventions using mixed WM training tasks, the single WM training paradigm is more time-saving and effective.

## Supporting information

S1 File Experiment data of Experiment 1. (RAR)

S2 File Experiment data of Experiment 2. (RAR)
